# Seropositivity of *Borrelia burgdorferi* s.l. in Germany—an analysis across four German National Cohort (NAKO) study sites

**DOI:** 10.1038/s41598-023-47766-6

**Published:** 2023-11-30

**Authors:** Max J. Hassenstein, Tobias Pischon, André Karch, Annette Peters, Tobias Kerrinnes, Henning Teismann, Alexandra Schneider, Sigrid Thierry, Ilais Moreno Velásquez, Jürgen Janke, Yvonne Kemmling, Stefanie Castell

**Affiliations:** 1 Department for Epidemiology, Helmholtz Centre for Infection Research (HZI), Braunschweig, Germany; 2 PhD Programme “Epidemiology”, Braunschweig-Hannover, Germany; 3 Max-Delbrueck-Center for Molecular Medicine in the Helmholtz Association (MDC), Molecular Epidemiology Research Group, Berlin, Germany; 4 Max-Delbrueck-Center for Molecular Medicine in the Helmholtz Association (MDC), Biobank Technology Platform, Berlin, Germany; 5 Berlin Institute of Health at Charité-Universitätsmedizin Berlin, Core Facility Biobank, Berlin, Germany; 6 Charité-Universitätsmedizin Berlin, corporate member of Freie Universität Berlin and Humboldt-Universität Zu Berlin, Berlin, Germany; 7 Institute of Epidemiology and Social Medicine, University of Münster, Münster, Germany; 8 Institute of Epidemiology, Helmholtz Zentrum München–German Research Center for Environmental Health (GmbH), Neuherberg, Germany; 9 Chair of Epidemiology, Institute for Medical Information Processing, Biometry and Epidemiology, Medical Faculty, Ludwig-Maximilians-Universität München, Munich, Germany; 10 Department of RNA-Biology of Bacterial Infections, Helmholtz Institute for RNA-Based Infection Research, Würzburg, Germany; 11 NAKO Studienzentrum, Klinik für Diagnostische und Interventionelle Radiologie und Neuroradiologie, Universitätsklinikum Augsburg, Augsburg, Germany; 12 TWINCORE, Centre for Experimental and Clinical Infection Research, a Joint Venture of the Hannover Medical School and Helmholtz Centre for Infection Research, 30625 Hannover, Germany

**Keywords:** Bacteria, Pathogens, Microbiology, Risk factors, Diagnostic markers

## Abstract

Lyme borreliosis (LB) is caused by the transmission of *Borrelia burgdorferi* s.l. from ticks to humans. Climate affects tick abundance, and climate change is projected to promote shifts in abundance in Europe, potentially increasing human exposure. We analyzed serum samples collected between the years 2014–2019 from German National Cohort (NAKO) participants at four study sites (Augsburg, Berlin, Hanover, Münster) for immunoglobulin G (IgG) and immunoglobulin M (IgM) antibodies using an enzyme‐linked immunosorbent assay (ELISA) and line blot immunoassay as confirmatory test for positive and equivocal ELISA samples. We reported crude and weighted seropositivity proportions for local estimates. We used mixed model analysis to investigate associated factors, such as age, sex, migration background, or animal contacts. We determined the serostatus of 14,207 participants. The weighted seropositivity proportions were 3.4% (IgG) and 0.4% (IgM) in Augsburg, 4.1% (IgG) and 0.6% (IgM) in northern Berlin, 3.0% (IgG) and 0.9% (IgM) in Hanover, and 2.7% (IgG) and 0.6% (IgM) in Münster. We found higher odds for IgG seropositivity with advancing age (*p* < 0.001), among males compared to females (*p* < 0.001) and reduced odds among participants with migration background compared to those without (*p* = 0.001). We did not find evidence for an association between serostatus and depression, children within the household, or animal contact, respectively. We found low seropositivity proportions and indications of differences across the study locations, although between-group comparisons did not yield significant results. Comparisons to earlier research are subject to important limitations; however, our results indicate no major increases in seropositivity over time. Nevertheless, monitoring of seropositivity remains critical in light of potential climate-related *Borrelia* exposure.

## Introduction

In Europe, Lyme borreliosis (LB) is the most frequent tick-borne disease caused by *Borrelia burgdorferi* sensu lato (*B. burgdorferi* s.l.). Climatic factors affect the geographical spread of *Ixodes ricinus (I. ricinus)*, the primary vector of *B. burgdorferi* s.l. in Europe^1^. Due to climate change-related temperature increases and humidity alterations, *I. ricinus* expanded its territory while also showing prolonged seasonal activity^2^, implying an increased potential risk for human exposure to ticks.

Especially in central and northern Europe, reported LB cases have increased within the past two decades^3^. However, LB is a notifiable disease in selected, but not all European countries^4^. In Germany, LB is partially notifiable in nine of sixteen federal states; notification data from these states indicate a varying but steady incidence by region for the years 2013–2017, ranging from 26 to 41 cases per 100,000 persons^5^, while nation-wide health insurance data from 2019 indicated an incidence of 179 cases of LB per 100,000 insured persons in Germany^6^.

Two recent local serological surveys conducted in Hanover, Northern Germany (2014–2018)^7^, and in Bonn in Western Germany (2018–2020)^8^, respectively, found no increases in seropositivity proportions compared to earlier investigations (1997–1999 and 2008–2011, respectively)^9^, despite increases in tick density in at least one German region (Siebengebirge near Bonn). The data for Hanover is included in this work with a different research focus.

Our work aims (1) to estimate the local seropositivity proportion of antibodies against *B. burgdorferi* s.l. among the general population of four different regions in Germany based on a nation-wide population-based cohort study (Augsburg in southern, Berlin North in Eastern-, Hanover in Northern-, and Münster in Western Germany) with—for the first time—high accuracy due to large sample size; (2) to compare seropositivity using identical age- and sex population weights; and (3) to investigate risk factors for seropositivity across regions. The data create a base for future comparisons regarding potentially increasing human *B. burgdorferi* s.l. exposure and enable investigations of seroconversion and –reversion within a large and ongoing cohort.

## Methods

The German National Cohort (NAKO) is a prospective population-based cohort study with 205,415 baseline participants examined across 18 study sites^10^. All potential NAKO-participants were randomly drawn from population registries. Subjects were eligible for participation when aged between 20 and 69 years at sampling date, provided written informed consent, had their primary residence within a regionally limited catchment area corresponding to the responsible NAKO study site, and were of sufficient health for study participation on site. Participants aged 40 years and above were oversampled. Detailed information is provided by Peters et al.^10^. All participants provided written informed consent. In our investigation, we randomly selected baseline serological samples from the urbanized sites Augsburg, Berlin North, Münster, and used all eligible samples from Hanover. The Hanover data were reported before^7^ with a different research focus. We performed stratified random sampling for the three other study sites after restricting to those with: available data for risk factor evaluation (if possible), focusing on a target distribution of 50% males and a distribution of age groups corresponding to the original NAKO recruitment, i.e., an oversampling of the age groups ≥ 40 years.

A laboratory (DIN EN ISO accredited, ISO 9001 certified) screened all samples for immunoglobulin G (IgG) and immunoglobulin M (IgM) *B. burgdorferi* s.l. antibodies using an enzyme‐linked immunosorbent assay (ELISA). Positive and equivocal ELISA samples underwent line blot immunoassay for confirmation (test kit information: Table S1).

In the primary analysis, we considered a sample with positive or equivocal ELISA and positive line blot result as a seropositive (classification according to the microbiologic-infectiologic quality standard MIQ12). For the secondary analysis to allow comparability with two previous studies, we considered a positive ELISA sample with subsequent positive or equivocal line blot or an equivocal ELISA with a positive line blot as positive ^9^. We classified each sample for IgG and IgM serostatus based on these two schemes. To estimate general population seropositivity, we weighted our sample with the respective local age and sex distribution based on the 2020 update of the 2011 census (www.destatis.de). For comparison with earlier estimates, we additionally weighted by the age- and sex distributions of the underlying study populations of the German National Health Interview and Examination Survey 1998 (BGS98, 1997–1999) and the German Health Interview and Examination Survey for Adults (DEGS, 2008–2011)^9^. Due to low total number of IgM-positive samples, we restricted further analyses to IgG antibodies. We used the χ^2^-test for trend in proportions to investigate a potential seropositivity trend with age, and with increasing education level. Then, we used the χ^2^-test (two-sided) to test for differences in German standard-population-weighted (2020 update of the 2011 census) seropositivity proportions between the study sites, and used post-hoc pairwise comparisons between the individual centers using the Fisher’s exact test (two-sided) with Bonferroni correction.

To evaluate potential risk factors for seropositivity, we used logistic mixed model analysis, with study centers as the clusters. We constructed models to estimate odds ratios (OR) for IgG seropositivity; in model 1, as a function of sex, age, education level, migration background, depression (score ≥ 10 of the 9-question Patient Health Questionnaire, PHQ-9), children within the household; in model 2, as a function of all variables from model 1 and additionally animal-related variables (having a particular pet vs. no pet) due to their restricted availability by NAKO design. We controlled for education level and migration background in our models to add additional evidence to recent conflicting findings concerning the role of socioeconomic factors concerning seropositivity^7, 8^. Due to the indication of a potential association of seropositivity with depressive symptomatology ^7^, we also added depression as control variable to obtain further insight. To provide insights into potentially altered tick exposure with children living in the same household, e.g., by altered outdoor activities^8^, we added this variable to our models as control variable.

We considered a significance level of 5% to determine statistical significance. All analysis and visualization was conducted in R version 4.1.2. We used the package “survey” to apply weights, “gtsummary” to support table creation, “ggplot” for visualization, and the glmer()-function from “lme4” to fit the mixed model. Finally, we used the “DHARMa”-package for further inspection of the model, e.g., residual analysis.

### Ethics approval

The Bavarian Medical Association (“Bayerische Landesärztekammer”) approved all NAKO-related human examinations as the central ethics committee [13023, 13031]. Local medical associations additionally approved the examinations. Our study respects all national laws and the 1975 Declaration of Helsinki in the current version.

## Results

Blood samples of 14,207 participants were available for our analysis. The participants were aged 20–74 years, with a median age of 50; 50.0% were male, 55.6% reported high education according to ISCED97, and 18.0% had a migration background. The overall crude IgG-seropositivity proportion was 3.4%.

Generally, we found an increasing trend concerning crude seropositivity with advancing age (*p* < 0.001); however, differences were only significant when comparing the older age group 60–69 (5.2%; 95% confidence interval (CI) 4.5–5.9%) to the younger age group 20–29 (2.6%; 95%-CI 1.8–3.5%) (Table 1). Overall, males had higher seropositivity (4.8%; 95%-CI 4.3–5.3%) than females (2.0%; 95%-CI 1.6–2.3%). Seropositivity increased with education: 1.3% (95%-CI 0.0–2.2%) in the lowest group and 3.9% (95%-CI 3.4–4.3) in the highest education group across all sites (p < 0.001). Participants without a migration background had higher seropositivity (3.7%; 95%-CI 3.4–4.0%) than those with a migration background (2.0%; 95%-CI 1.5—2.6). We did not observe differences in seropositivity between participants with depression (3.0%; 95%-CI 2.2–3.8%) or without depression (3.5%; 95%-CI 3.1–3.8%) and between participants with pets (dogs: 2.3%; 95%-CI 0.8–3.8%) compared to those without pets (3.9%; 95%-CI 3.1–4.8%).

**Table 1:** Population characteristics and crude proportions of Immunoglobulin G antibody detection against *Borrelia burgdorferi* s.l.

	Crude Immunoglobulin G seropositivity against *B. burgdorferi* s.l., proportion (%, 95% confidence interval)				
	Overall (N = 14,207)	Augsburg (n = 2587)	Berlin (north) (n = 1250)	Hanover (n = 8007)	Münster (n = 2363)
Overall	482/14,207 (3,4; 3,1–3,7)	99/2587 (3.8; 3.1–4.6)	58/1250 (4.6; 3.5–5.8)	252/8007 (3.1; 2.8–3.5)	73/2363 (3.1; 2.4–3.8)
Age					
20–29	38/1442 (2.6; 1.8–3.5)	5/259 (1.9; 0.3–3.6)	3/118 (2.5; 0.0–5.4)	23/831 (2.8; 1.7–3.9)	7/234 (3.0; 0.8–5.2)
30–39	25/1394 (1.8; 1.1–2.5)	5/262 (1.9; 0.3–3.6)	4/128 (3.1; 0.1–6.1)	14/769 (1.8; 0.9–2.8)	2/235 (0.9; 0.0–2)
40–49	82/3783 (2.2; 1.7–2.6)	13/691 (1.9; 0.9–2.9)	12/349 (3.4; 1.5–5.4)	47/2099 (2.2; 1.6–2.9)	10/644 (1.6; 0.6–2.5)
50–59	139/3777 (3.7; 3.1–4.3)	35/697 (5.0; 3.4–6.6)	11/328 (3.4; 1.4–5.3)	67/2117 (3.2; 2.4–3.9)	26/635 (4.1; 2.6–5.6)
60–69	188/3618 (5.2; 4.5–5.9)	41/678 (6.0; 4.3–7.8)	28/327 (8.6; 5.5–11.6)	91/1998 (4.6; 3.6–5.5)	28/615 (4.6; 2.9–6.2)
70–74	10/193 (5.2; 2.1–8.3)	–	–	10/193 (5.2; 2.1–8.3)	–
Gender					
Male	343/7108 (4.8; 4.3–5.3)	65/1306 (5.0; 3.8–6.2)	41/623 (6.6; 4.6–8.5)	181/3991 (4.5; 3.9–5.2)	56/1188 (4.7; 3.5–5.9)
Female	139/7099 (2.0; 1.6–2.3)	34/1281 (2.7; 1.8–3.5)	17/627 (2.7; 1.4–4)	71/4016 (1.8; 1.4–2.2)	17/1175 (1.4; 0.8–2.1)
Education (ISCED97)					
Low or ongoing	8/627 (1.3; 0.4–2.2)	1/135 (0.7; 0.0–2.2)	0/30 (0.0; 0.0–0.0)	4/377 (1.1; 0.0–2.1)	3/85 (3.5; 0.0–7.5)
Medium	171/5475 (3.1; 2.7–3.6)	48/1347 (3.6; 2.6–4.6)	22/484 (4.5; 2.7–6.4)	74/2778 (2.7; 2.1–3.3)	27/866 (3.1; 2.0–4.3)
High	294/7631 (3.9; 3.4–4.3)	50/1105 (4.5; 3.3–5.8)	36/736 (4.9; 3.3–6.4)	165/4379 (3.8; 3.2–4.3)	43/1411 (3; 2.2–3.9)
Missing	–	–	–	9/473 (1.9; 0.7–3.1)	0/1 (0; 0–0)
Migration background					
No	430/11,640 (3.7; 3.4–4.0)	90/2057 (4.4; 3.5–5.3)	57/1172 (4.9; 3.6–6.1)	217/6388 (3.4; 3–3.8)	66/2023 (3.3; 2.5–4.0)
Yes	52/2563 (2.0; 1.5–2.6)	9/530 (1.7; 0.6–2.8)	1/78 (1.3; 0.0–3.8)	35/1615 (2.2; 1.5–2.9)	7/340 (2.1; 0.5–3.6)
Missing	–	–	–	0/4 (0.0; 0.0–0.0)	–
Children within the household					
No	413/11,408 (3.6; 3.3–4.0)	89/2024 (4.4; 3.5–5.3)	48/984 (4.9; 3.5–6.2)	213/6530 (3.3; 2.8–3.7)	63/1870 (3.4; 2.6–4.2)
Yes	69/2788 (2.5; 1.9–3.1)	10/562 (1.8; 0.7–2.9)	10/266 (3.8; 1.5–6.0)	39/1468 (2.7; 1.8–3.5)	10/492 (2.0; 0.8–3.3)
Missing	–	0/1 (0.0; 0.0–0.0)	–	0/9 (0.0; 0.0–0.0)	0/1 (0.0; 0.0–0.0)
Depression (PHQ-9)					
No	431/12,492 (3.5; 3.1–3.8)	95/2394 (4.0; 3.2–4.8)	53/1168 (4.5; 3.3–5.7)	215/6696 (3.2; 2.8–3.6)	68/2234 (3.0; 2.3–3.8)
Yes	51/1715 (3.0; 2.2–3.8)	4/193 (2.1; 0.1–4.1)	5/82 (6.1; 0.9–11.3)	37/1311 (2.8; 1.9–3.7)	5/129 (3.9; 0.5–7.2)
Animals within household ^1^					
None	77/1957 (3.9; 3.1–4.8)	23/577 (4.0; 2.4–5.6)	6/133 (4.5; 1–8)	35/1016 (3.4; 2.3–4.6)	13/231 (5.6; 2.7–8.6)
Dog	9/386 (2.3; 0.8–3.8)	4/152 (2.6; 0.1–5.2)	2/31 (6.5; 0.0–15.1)	2/155 (1.3; 0.0–3.1)	1/48 (2.1; 0.0–6.1)
Cat	13/495 (2.6; 1.2–4.0)	6/245 (2.4; 0.5–4.4)	2/50 (4.0; 0.0–9.4)	4/161 (2.5; 0.1–4.9)	1/39 (2.6; 0.0–7.5)
Other	3/161 (1.9; 0.0–4.0)	1/62 (1.6; 0.0–4.7)	1/15 (6.7; 0.0–19.3)	0/66 (0.0; 0.0–0.0)	1/18 (5.6; 0.0–16.1)

ISCED = International Standard Classification of Education; PHQ-9 = Patient Health Questionnaire 9; We defined seropositivity as positive or equivocal ELISA screening test with positive confirmatory line blot immunoassay; proportions presented with two-sided 95%-confidence interval (Wald); ^1^ animal-related questions were part of the extended examination program of the NAKO and, therefore, only available for selected participants (n = 2878).

**Figure 1:**
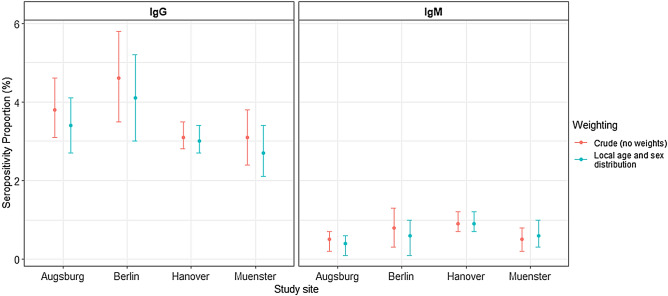
Crude (red) and weighted (blue) seropositivity proportions with 95% confidence intervals of IgG and IgM antibodies against *Borrelia burgdorferi s.l.* IgG = Immunoglobulin G; IgM = Immunoglobulin M; We defined seropositivity as positive or equivocal ELISA screening test with positive confirmatory line blot immunoassay. We weighted our sample with the respective local age and sex distributions.

Local population-weighted point seropositivity varied by location (Fig. 1, Table S2). Participants from Berlin had the highest weighted IgG seropositivity proportion (4.1%, 95%-CI 3.0–5.2%), while those from Münster had the lowest weighted proportion (2.7%, 95%-CI 2.1–3.4). The CI of seropositivity proportions overlapped between the four sites. However, the χ^2^-test indicated overall differences in proportions for estimates weighted by nation-wide age and sex population proportions from 2020 (*p* = 0.04). The pairwise comparisons with Fisher's exact test did not yield significant results at the 95% significance level. Table S3 reports weighted seropositivity using the age- and sex distribution of earlier studies^9^.

From logistic mixed-model analysis, we found that every 10-year increase in age was associated with 1.26-fold (95%-CI 1.16–1.38) the odds for IgG-seropositivity (Table 2). Men had 2.54 times (95%-CI 2.05–3.13) the odds of being IgG-seropositive compared to females. Participants with a migration background had 0.56 (95%-CI 0.41–0.78) times the odds for a positive IgG-serostatus compared to participants with no migration background. We did not find evidence for an association of IgG serostatus with education level, depression on a binary scale (PHQ-9), children within the household (any versus none), or any current or previous animal contacts, respectively.

**Table 2:** Model-based odds ratios from mixed-effects logistic regression for positive immunoglobulin G serostatus against *Borrelia burgdorferi* s.l.

	Model 1 (n = 13,024)			Model 2 (n = 2878)		
	OR	95%-CI	*p*	OR	95%-CI	*p*
Age in 10-year increments	1.26	1.16–1.38	< 0.001	1.29	1.07–1.54	0.009
Sex						
Female	Ref	Ref	Ref	Ref	Ref	Ref
Male	2.54	2.05–3.13	< 0.001	2.25	1.44–3.51	< 0.001
Education (ISCED97)						
Low or ongoing	0.57	0.26–1.23	0.150	–	–	–
Medium	Ref	Ref	Ref	–	–	–
High	1.12	0.91–1.37	0.283	–	–	–
Migration background						
No	Ref	Ref	Ref	Ref	Ref	Ref
Yes	0.56	0.41–0.78	0.001	0.28	0.11–0.69	0.006
Depression (PHQ-9)						
No	Ref	Ref	Ref	Ref	Ref	Ref
Yes	0.80	0.51–1.25	0.327	0.85	0.31–2.37	0.761
Children within the household						
No	Ref	Ref	Ref	Ref	Ref	Ref
Yes	0.77	0.58–1.02	0.067	1.16	0.68–2.00	0.587
Animals within household						
None	–	–	–	Ref	Ref	Ref
Dog	–	–	–	0.63	0.31–1.28	0.206
Cat	–	–	–	0.76	0.41–1.38	0.362
Other	–	–	–	0.55	0.17–1.78	0.319

OR = Odds Ratio; ISCED = International Standard Classification of Education; PHQ-9 = Patient Health Questionnaire 9; We defined seropositivity as positive or equivocal ELISA screening test with positive confirmatory line blot immunoassay; Model 1 contained all study participants; Model 2 contained only participants from the extended module, for which variables on animal contacts were available; in model 2, we excluded education as independent variable due to low person count in the low education group.

## Discussion

We conducted the largest-ever German multi-center serological survey for *B. burgdorferi* s.l. in Germany. We compared seropositivity estimates of four German regions representing the four cardinal directions. The crude proportion of seropositive participants slightly varied by study location. We estimated the local seropositivity to lie between 4.1% in Berlin (95%-CI 3.0–5.2%) and 2.7% in Münster (95%-CI 2.1–3.4).

The comparison of our findings to earlier studies is subject to critical limitations due to differences in the sampling methodology. We could not account for factors such as the degree of rurality or other living conditions. However, when classifying our samples like in^9^ and weighting them by age and sex for comparability to two earlier studies^9^ based on BGS98 and DEGS, we did not find any indication for relevant increases in seropositivity. Seropositivity in Augsburg was lower in our study when compared to BGS98 (6.1%, 95%-CI 5.1–7.0% vs. 10.4%, 95%-CI 7.5–13.4%) and DEGS estimates (6.5%, 95%-CI 5.5–7.4% vs. 12.1, 95%-CI 8.8–15.4%) for Bavaria. Seropositivity for Hanover was comparable with Lower-Saxony estimates in BGS98 (4.9%, 95%-CI 4.5–5.4% vs. 7.4%, 95%-CI 5.1–9.6%), but lower when compared to DEGS (5.3%, 95%-CI 4.8–5.8% vs. 9.1%, 95%-CI 6.7–11.4%). For northern Berlin, seropositivity was comparable to BGS98 jointly reported estimates for Brandenburg, Mecklenburg-Vorpommern, and Saxony-Anhalt (5.0%, 95%-CI 3.8–6.3% vs. 7.2%, 95%-CI 5.8–8.7%). Similarly, seropositivity was comparable when comparing northern Berlin seropositivity estimates to DEGS estimates (5.6%, 95%-CI 4.3–6.9% vs. 9.3%, 95%-CI 6.8–11.9). When considering the seropositivity proportions for Münster, we found a similar seropositivity proportion compared to earlier BGS98-estimates for North Rhine-Westphalia (5.2%, 95%-CI 4.3–6.1% vs. 7.4%, 95%-CI 5.1–9.6). However, seropositivity was lower than DEGS estimates (5.5%, 95%-CI 4.5–6.4 vs. 9.1%, 95%-CI 6.7–11.4).

All four study sites recruited participants from predominantly urban areas, which may have resulted in reduced seropositivity proportion compared to earlier investigations, which included a higher proportion of rural participants. Within DEGS, participants living in municipalities with less than 5000 inhabitants had twice the chance for being seropositive compared with those with more than 100,000 inhabitants^11^, potentially due to higher exposure to green-space areas and, therefore, ticks. Notification data from Bavaria and Berlin supports this by showing that, compared to the surrounding region, Berlin and Augsburg had lower incidences^5^.

Our results confirm advancing age and male sex as risk factors for positive antibody detection. As found in earlier studies, adults with a migration background were less likely to be seropositive^11, 12^. We did not find conclusive evidence for an association between educational level, current depression (PHQ-9 score ≥ 10)**,** or previous or current animal contact with serostatus, respectively. Our results from multivariable modelling concerning educational level are in line with previous studies, which also found no association between socio-economic status and serostatus^9^, but opposes recent findings from a population-based cohort in central Bonn, in which highly educated individuals had higher chances for positive serostatus compared to individuals with medium-level education^8^. The conflicting findings might be explainable by the availability or use of different outdoor spare time activities in different urban regions. Additional investigations including higher proportions of participants with lower and intermediate education are required to complement this discussion. In contrast to a previous study using the original PHQ-9-scores as a proxy for depression diagnosis^7^, we did not find evidence for an association between serostatus and depression on a binary scale.

Our findings did not support an association between animal contacts and seropositivity, which is in line with earlier findings among adults^11^, but contrasts a previous finding^12^, which found higher chances for seropositivity among children living in households with any pet vs. no pet or with a cat vs. no cat, respectively.

Our work has several limitations. Most samples considered in our regression analysis originate from the study center in Hanover. Hence, our analysis is predominantly driven by this single-site data. Furthermore, the use of test kits with differing specificity, sensitivity, and considered *Borrelia* strains between the studies hamper the comparability of seropositivity proportions of our study with previous analyses. A long-term cohort like NAKO can overcome this in the future by providing the basis for a harmonized longitudinal seropositivity analysis. The small number of seropositive individuals in the low education group and the groups of individuals with animals in the household may have resulted in low statistical power and, thus, the inability to identify a potential association in the regression models.

In conclusion, we found low seropositivity across four study locations across Germany, with minor indications of differences between sites. Our seropositivity estimates of the years 2014–2019 for four predominantly urban regions correspond to previous estimates for 1997–1999 and 2008–2011, respectively, indicating no considerable increases of seropositivity over time. However, comparisons to earlier estimates suffer from limitations. Even in urbanized regions, potential climate-change-related shifts in tick exposure may increase infections and, therefore, non-urgent follow-up may be conducted in future waves of the NAKO cohort study. Our findings underpin previously suggested risk factors for seropositivity, with advancing age and male sex as the most critical risk factors. Migration status appears as an additional factor of lower seropositivity since many persons migrating to Germany may come from countries with little or no *Borrelia* exposure.

### Supplementary Information

Supplementary Information.

## Data Availability

The German National Cohort (NAKO) data is not openly available due to data protection measures. However, scientists can apply for data access following the official usage regulations and upon formal request to the NAKO use and access committee (https://transfer.nako.de/).

## References

[1] Steere AC, Strle F, Wormser GP, Hu LT, Branda JA, Hovius JWR (2016). Lyme borreliosis. Nat. Rev. Dis. Prim..

[2] Estrada-Peña A, Fernández-Ruiz N (2020). A retrospective assessment of temperature trends in northern Europe reveals a deep impact on the life cycle of *Ixodes ricinus* (Acari: Ixodidae). Pathogens.

[3] Vandekerckhove O, de Buck E, van Wijngaerden E (2021). Lyme disease in Western Europe: An emerging problem? A systematic review. Acta Clin. Belg..

[4] Steinbrink A, Brugger K, Margos G, Kraiczy P, Klimpel S (2022). The evolving story of *Borrelia burgdorferi* sensu lato transmission in Europe. Parasitol. Res..

[5] Enkelmann J, Böhmer M, Fingerle V, Siffczyk C, Werber D, Littmann M (2018). Incidence of notified *Lyme borreliosis* in Germany, 2013–2017. Sci. Rep..

[6] Akmatov MK, Holstiege J, Dammertz L, Heuer J, Kohring C, Lotto-Batista M (2022). Epidemiology of *Lyme borreliosis* based on outpatient claims data of all people with statutory health insurance, Germany, 2019. Euro Surveill..

[7] Hassenstein MJ, Janzen I, Krause G, Harries M, Melhorn V, Kerrinnes T (2022). Seroepidemiology of *Borrelia burgdorferi* s.l. among German national cohort (NAKO) participants, Hanover. Microorganisms.

[8] Coors A, Hassenstein MJ, Krause G, Kerrinnes T, Harries M, Breteler MMB, Castell S (2022). Regional seropositivity for *Borrelia burgdorferi* and associated risk factors: findings from the Rhineland Study, Germany. Parasit. Vectors.

[9] Woudenberg T, Böhm S, Böhmer M, Katz K, Willrich N, Stark K (2020). Dynamics of *Borrelia burgdorferi*-specific antibodies: Seroconversion and seroreversion between two population-based, cross-sectional surveys among adults in Germany. Microorganisms.

[10] Peters A, Greiser KH, Göttlicher S, Ahrens W, Albrecht M, Bamberg F (2022). Framework and baseline examination of the German National Cohort (NAKO). Eur. J. Epidemiol..

[11] Wilking H, Fingerle V, Klier C, Thamm M, Stark K (2015). Antibodies against *Borrelia burgdorferi* sensu lato among Adults, Germany, 2008–2011. Emerg. Infect. Dis..

[12] Dehnert M, Fingerle V, Klier C, Talaska T, Schlaud M, Krause G (2012). Seropositivity of Lyme borreliosis and associated risk factors: a population-based study in children and adolescents in Germany (KiGGS). PLoS One.

