# Supplementary material for: Seropositivity of Borrelia burgdorferi s.l. in Germany—an analysis across four German National Cohort (NAKO) study sites
Source: Sci Rep. 2023 Nov 30;13:21087. doi: 10.1038/s41598-023-47766-6 (PMC10689756; doi:10.1038/s41598-023-47766-6)
Supplement: Supplementary file 1 — Supplementary Information. [file 41598_2023_47766_MOESM1_ESM.docx]

**Supplementary Information**

**European Journal of Epidemiology**

**Seropositivity of *Borrelia burgdorferi* s.l. in Germany – an analysis across four German National Cohort (NAKO) study sites**

Max J Hassenstein^1,2^, Tobias Pischon^3,4,5,6^, André Karch^7^, Annette Peters^8,9^, Tobias Kerrinnes^10^, Henning Teismann^7^, Alexandra Schneider^8^, Sigrid Thierry ^8,11^, Ilais Moreno Velásquez^3^, Jürgen Janke^3,4^, Yvonne Kemmling^1^, Stefanie Castell^1,12, *^

^1^ Department for Epidemiology, Helmholtz Centre for Infection Research (HZI), Braunschweig, Germany

^2^ PhD Programme “Epidemiology” Braunschweig-Hannover, Germany

^3^ Max-Delbrueck-Center for Molecular Medicine in the Helmholtz Association (MDC), Molecular Epidemiology Research Group, Berlin, Germany

^4^ Max-Delbrueck-Center for Molecular Medicine in the Helmholtz Association (MDC), Biobank Technology Platform, Berlin, Germany

^5^ Berlin Institute of Health at Charité - Universitätsmedizin Berlin, Core Facility Biobank, Berlin, Germany

^6^ Charité - Universitätsmedizin Berlin, corporate member of Freie Universität Berlin and Humboldt-Universität zu Berlin, Berlin, Germany

^7^ Institute of Epidemiology and Social Medicine, University of Münster, Münster, Germany

^8^ Institute of Epidemiology, Helmholtz Zentrum München – German Research Center for Environmental Health (GmbH), Neuherberg, Germany

^9^ Chair of Epidemiology, Institute for Medical Information Processing, Biometry and Epidemiology, Medical Faculty, Ludwig-Maximilians-Universität München, Munich, Germany

^10^ Department of RNA-Biology of Bacterial Infections, Helmholtz Institute for RNA-Based Infection Research, Würzburg, Germany

^11^ NAKO Studienzentrum, Klinik für Diagnostische und Interventionelle Radiologie und Neuroradiologie, Universitätsklinikum Augsburg

^12^ TWINCORE, Centre for Experimental and Clinical Infection Research, a Joint Venture of the Hannover Medical School and Helmholtz Centre for Infection Research, 30625 Hannover, Germany

*** Corresponding author. Email: Stefanie.Castell@helmholtz-hzi.de**

**Table S1** Test kits used for screening and confirmatory testing of antibody presence against *Borrelia burgdorferi* s.l.

| Antibody type | Test type | Test kit | Manufacturer | Sensitivity^1^ | Specificity^1^ | Strains |
| --- | --- | --- | --- | --- | --- | --- |
| Immunoglobulin G | Screening test, ELISA | *Borrelia afzelii* and VlsE IgG Europe ELISA | Virotech Diagnostics GmbH | >99% | 97% | - Zs7 (B. burgdorferi) - PBr (B. garinii) - Pko (B. afzelii) |
| Immunoglobulin M |  | *Borrelia afzelii* IgM ELISA |  |  | 98.8% |  |
| Immunoglobulin G | Confirmatory test, line blot | WE225 Borrelia Europe plus TpN17 LINE IgG | Virotech Diagnostics GmbH | >99.9% | 98% | - OpsC (p23) (B. afzelii PKo) - VlsE recombinant (B. burgdorferi B31) - p39 (BmpA) recombinant (B. afzelii PKo) - DbpA (Pko) and DbpA (PBi, PBr, A14 S) recombinant (B. bavariensis PBi, B. garinii PBr) - p58 (OppA‐2) recombinant (B. bavariensis PBi) - p83/100 recombinant (B. afzelii PKo) |
| Immunoglobulin M |  | WE224 Borrelia Europe LINE IgM |  |  |  |  |
| IgG = Immunoglobulin G; IgM = Immunoglobulin M; ELISA = enzyme-linked immunosorbent assay;  1 In brief and with reference to the information provided by the manufacturer [1-3], sensitivity and specificity were determined by testing clinically characterized sera. More information can be obtained from the original test kit documentations provided by the manufacturer [1-3]. | | | | | | |

**Table S2** Crude and weighted seropositivity proportions with 95% confidence interval of IgG and IgM antibodies against *Borrelia burgdorferi* s.l. For weighting, we used local age and sex distributions of the general population.

|  | Augsburg | | Berlin | | Hanover, as also published in [4] | | Münster | |
| --- | --- | --- | --- | --- | --- | --- | --- | --- |
|  | Proportion (%, 95% confidence interval) | | | | | | | |
|  | Crude | Weighted | Crude | Weighted | Crude | Weighted | Crude | Weighted |
| IgG: ELISA* | 9.7 (8.6-10.8) | 8.7 (7.6-9.9) | 5.8 (4.5-7.1) | 4.9 (3.7-6.1) | 7.0 (6.5-7.6) | 6.8 (6.3-7.4) | 9.1 (7.9-10.2) | 8.8 (7.6-9.9) |
| IgG: RKI** | 6.9 (5.9-7.9) | 6.2 (5.3-7.2) | 6.1 (4.8-7.4) | 5.0 (3.8-6.3) | 5.4 (4.9-5.9) | 5.2 (4.7-5.6) | 5.8 (4.9-6.8) | 5.1 (4.2-6.0) |
| IgG: MIQ12*** | 3.8 (3.1-4.6) | 3.4 (2.7-4.1) | 4.6 (3.5-5.8) | 4.1 (3.0-5.2) | 3.1 (2.8-3.5) | 3.0 (2.7-3.4) | 3.1 (2.4-3.8) | 2.7 (2.1-3.4) |
| IgM: ELISA* | 0.6 (0.3-0.9) | 0.5 (0.3-0.8) | 1.2 (0.6-1.8) | 1.0 (0.4-1.5) | 2.0 (1.7-2.3) | 2.1 (1.8-2.4) | 1.0 (0.6-1.4) | 1.0 (0.6-1.4) |
| IgM: RKI** | 0.7 (0.4-1.0) | 0.6 (0.3-0.9) | 0.8 (0.3-1.3) | 0.6 (0.1-1.0) | 1.3 (1.1-1.6) | 1.4 (1.2-1.7) | 0.6 (0.3-1) | 0.8 (0.4-1.1) |
| IgM: MIQ12*** | 0.5 (0.2-0.7) | 0.4 (0.1-0.6) | 0.8 (0.3-1.3) | 0.6 (0.1-1.0) | 0.9 (0.7-1.2) | 0.9 (0.7-1.2) | 0.5 (0.2-0.8) | 0.6 (0.3-1.0) |
| IgG = Immunoglobulin G; IgM = Immunoglobulin M; ELISA = enzyme-linked immunosorbent assay; RKI = Robert Koch Institute; * seropositivity is defined by a positive ELISA result; ** seropositivity defined as positive ELISA screening test and positive or equivocal line blot or equivocal ELISA screening test and positive confirmatory line blot immunoassay [2]; *** we defined seropositivity as positive or equivocal ELISA screening test with positive confirmatory line blot immunoassay; proportions presented with two-sided 95%-confidence interval (Wald); we weighted our sample with the respective local age and sex distribution (www.destatis.de); Note: Usually, the ELISA-only classification (*) is the most sensitive. However, due to a higher share of ELISA-equivocal samples in our study, the second most sensitive classification (**) showed higher proportions of overall seropositivity in a few instances. | | | | | | | | |

**Table S3** Weighted seropositivity percentages with 95% confidence interval of IgG antibodies against *Borrelia burgdorferi* s.l. for comparison with earlier investigation

|  | Augsburg | | Berlin | | Hanover | | Münster | |
| --- | --- | --- | --- | --- | --- | --- | --- | --- |
|  | Weighted % (95% confidence interval) | | | | | | | |
|  | BGS98 | DEGS | BGS98 | DEGS | BGS98 | DEGS | BGS98 | DEGS |
| IgG: ELISA* | 8.5 (7.4-9.6) | 9.2 (8.0-10.3) | 4.8 (3.6-6.0) | 5.4 (4.1-6.6) | 6.6 (6.0-7.1) | 7.0 (6.4-7.5) | 8.8 (7.7-10) | 8.8 (7.7-9.9) |
| IgG: RKI** | 6.1 (5.1-7.0) | 6.5 (5.5-7.4) | 5.0 (3.8-6.3) | 5.6 (4.3-6.9) | 4.9 (4.5-5.4) | 5.3 (4.8-5.8) | 5.2 (4.3-6.1) | 5.5 (4.5-6.4) |
| IgG: MIQ12*** | 3.2 (2.6-3.9) | 3.6 (2.8-4.3) | 4.0 (2.9-5.1) | 4.3 (3.2-5.4) | 2.9 (2.5-3.3) | 3.1 (2.7-3.5) | 2.6 (2.0-3.3) | 2.9 (2.2-3.6) |
| IgG = Immunoglobulin G; RKI = Robert Koch Institute; BGS98 = German National Health Interview and Examination Survey 1998; DEGS = German Health Interview and Examination Survey for Adults; For comparability, we weighted our sample with the age- and sex distributions of the underlying study population BGS98 and DEGS [2]; * seropositivity is defined by a positive ELISA result; ** seropositivity defined as positive ELISA screening test and positive or equivocal line blot or equivocal ELISA screening test and positive confirmatory line blot immunoassay [5]; *** we defined seropositivity as positive or equivocal ELISA screening test with positive confirmatory line blot immunoassay | | | | | | | | |

**References**

1. VIROTECH Diagnostics GmbH. Diagnostic Test Kit for Borrelia (Borrelia + VlsE IgG ELISA). REV 17. 2019
2. VIROTECH Diagnostics GmbH. Diagnostic Test Kit for Borrelia (Borrelia IgM ELISA). REV 17. 2019
3. VIROTECH Diagnostics GmbH. Diagnostic Test Kit for Borrelia (Borrelia EU IgG LINE-32; Borrelia EU IgG LINE-96). REV 17. 2018
4. Hassenstein MJ, Janzen I, Krause G, Harries M, Melhorn V, Kerrinnes T, et al. Seroepidemiology of Borrelia burgdorferi s.l. among German National Cohort (NAKO) Participants, Hanover. Microorganisms 2022. doi:10.3390/microorganisms10112286.
5. Woudenberg T, Böhm S, Böhmer M, Katz K, Willrich N, Stark K, et al. Dynamics of Borrelia burgdorferi-Specific Antibodies: Seroconversion and Seroreversion between Two Population-Based, Cross-Sectional Surveys among Adults in Germany. Microorganisms 2020. doi:10.3390/microorganisms8121859.
